# Development of a DNA damage assay system using stable human hepatocytes

**DOI:** 10.1186/s41021-025-00347-7

**Published:** 2026-01-27

**Authors:** Masayuki Mishima, Kazuki Izawa, Masataka Tsuda, Yuichiro Higuchi, Shotaro Uehara, Hiroshi Suemizu, Kei-Ichi Sugiyama

**Affiliations:** 1 Division of Genome Safety Science, National Institute of Health Sciences (NIHS), 3-25-26 Tonomachi, Kawasaki-ku, Kawasaki, Kanagawa 210-9501 Japan; 2 Liver Engineering Laboratory, Department of Research for Humanized Model, Central Institute for Experimental Medicine and Life Science (CIEM), 3-25-12 Tonomachi, Kawasaki-ku, Kawasaki, Kanagawa 210-0821 Japan

**Keywords:** Human hepatocytes, Genotoxicity, γh2AX, Metabolic activation, Liver s9, HepaSH cells

## Abstract

**Background:**

Overcoming species differences in metabolism between humans and animals remains a critical challenge in toxicological studies. Rat liver S9 fraction has long been the gold standard for exogenous metabolic activation in in vitro genotoxicity tests. Experiences with human S9 or human primary hepatocytes have suggested that the human materials are unsuitable for standardized testing due to high variability. Nevertheless, there is growing interest in genotoxicity evaluation using metabolic systems that more closely mimic human physiology.

**Results:**

We developed an in-cell ELISA system to measure γH2AX as a DNA damage marker in stable human hepatocytes (γH2AX-SHE). HepaSH cells are consistently available human hepatocytes that stably express a range of metabolic enzymes and drug transporters in vitro. Due to their highly differentiated and non-proliferative nature, conventional genotoxicity endpoints such as micronuclei formation, chromosomal aberrations, or mutant colony assays are not applicable. We used γH2AX, a sensitive DNA damage marker, in this assay system. Indirect mutagens including benzo(a)pyrene, aristolochic acid, and 2-Amino-1-methyl-6-phenylimidazo(4,5-b)pyridine induced dose-dependent increases in γH2AX across all three HepaSH strains. Time-course analysis following benzo(a)pyrene exposure indicated that a treatment duration of 16 hours or longer was necessary to detect genotoxic responses. Prolonged exposure for 48 hours resulted in extensive cell death, which may interfere with γH2AX quantification.

**Conclusions:**

We demonstrated that γH2AX-SHE can serve as a valuable tool for detecting DNA damage under conditions that mimic human metabolic activity. Based on the findings in this study, we recommend the following assay conditions for γH2AX-SHE: a 24-hour treatment period, a DMSO concentration not exceeding 1%, and careful interpretation of positive responses observed at highly cytotoxic doses - defined as approximately less than 60% cell survival – as these may lack biological relevance.

## Introduction

Overcoming species differences in metabolism between humans and animals remains a critical challenge in toxicological studies. In the genotoxicity assessments, rat liver S9 fraction prepared from animals treated with enzyme-inducing chemicals like aroclor 1254 has long been considered the gold standard of exogenous metabolic activation in in vitro tests. Ames test and mammalian cell assays using rat S9 are widely accepted for regulatory purposes [1–4]. However, genotoxicity tests of certain compounds using alternative S9 sources have yielded divergent results [5–7]. An emerging topic of enhanced Ames test includes use of both rat and hamster S9 to improve sensitivity to nitrosamine compounds [8, 9]. There is growing interest in genotoxicity evaluation employing more human-like metabolic system.

Incorporating a human-mimicking metabolic activation system into an in vitro experiment remains a significant challenge, as currently available human hepatocytes or human S9 fractions are often unsuitable for standard tests due to substantial variability. Human hepatocytes cannot be obtained from healthy donors for experimental purposes. Factors such as underlying diseases requiring liver resection, medications administered to patients, and suboptimal handling of excised liver tissue under inadequate preservation conditions significantly affect hepatocyte activity. Therefore, we hesitate to regard primary human hepatocytes or human S9 as representative models of the human metabolic system.

Efforts to identify alternative cell sources to primary human hepatocytes have been ongoing. Hepatocyte-like cells derived from human induced pluripotent stem (iPS) cells have demonstrated the ability to detect metabolic-mediated toxicity [10–12]. However, genomic instability in iPS cells [13, 14] remains a fundamental concern for their use in genotoxicity assessment. Although the genomic stability of HepaSH cells has not been fully elucidated, they are expected to maintain typical stability comparable to primary hepatocytes cultures. Genetically engineered human cell lines expressing human cytochrome P450 (CYPs) enzymes have been developed [15–19]. These recombinant cells offer advantages of stable phenotype, high-level expression of target CYP species, and rapid proliferation. Those cells are particularly useful for testing compounds when the key CYP enzymes involved in their metabolism are known. For compounds with unknown metabolic pathways, cells expressing a broad spectrum of metabolic enzymes are desirable.

HepaSH cells [20] are commercially available human hepatocytes. Cryopreserved human primary hepatocytes are incubated and expanded in immunodeficient mice, then harvested and supplied as HepaSH cells. HepaSH cells stably express various metabolic enzymes and transporters for up to one month in vitro. The relative gene expression levels of C-reactive protein, a marker of inflammation or tissue injury, ranged 0 - 2.5 in primary human hepatocytes, and decreased to 0.5 or less in HepaSH cells [20], suggesting recovery of the original human hepatocytes from initial damage.

In this study, we developed a genotoxicity assay system with HepaSH cells. The cells were highly differentiated and non-proliferating, traditional genotoxicity biomarkers like micronuclei, chromosomal aberration or mutant colony formation cannot be used. We developed an in-cell ELISA system to measure γH2AX, a DNA damage marker, in HepaSH cells. After treatment with indirect genotoxicants which need metabolic activation to exhibit genotoxicity, γH2AX was dose dependently increased in HepaSH cells.

## Materials and Methods

### Ethics

In vivo procedures in this study were performed in strict accordance with the Guide for the Care and Use of Laboratory Animals of the Central Institute for Experimental Medicine and Life Science (CIEM) Japan in an exploratory manner. Use of the human materials were done in accordance of the Declaration of Helsinki and approved by the Institutional Ethics Committee of either NIHS or CIEM.

### Chemicals

Direct mutagens, mitomycin C (MMC, CASRN 50-07-7, Nacalai Tesque, Kyoto, Japan) and oxaliplatin (CASRN 63121-00-6, Fujifilm Wako, Osaka, Japan), metabolically activated mutagens, benzo(a)pyrene (BaP, CASRN 50-32-8, Sigma Aldrich, St. Louis, MO), aristolochic acid (AA, CASRN 10190-99-5, Sigma Aldrich, St. Louis, MO) and 2-amino-1-methyl-6-phenylimidazo(4,5-b)pyridine (PhIP, CASRN 105650-23-5, Fujifilm Wako), and non-mutagens, sodium dodecyl sulfate (SDS, CASRN 151-21-3, Fujifilm Wako, Osaka, Japan) and sodium chloride (CASRN 7647-14-5, Fujifilm Wako, Osaka, Japan), were used. The test compounds were dissolved in dimethyl sulfoxide (DMSO, Nacalai Tesque, Kyoto, Japan) or culture medium and appropriately diluted with culture medium.

### Cells

HepaSH cells prepared using commercially available cryopreserved hepatocytes derived from three human donors (HepaSH cells strain E, HepaSH-E; Female, Caucasian, 12 years old, HepaSH cells strain AD, HepaSH-AD; Male, Caucasian, 3 months old, and HepaSH cells strain L, HepaSH-L; Male, Caucasian, 5 years old) were used. The cells were isolated from chimeric mice in CIEM and maintained through the experimental duration in NIHS under the culture conditions previously described [20, 21]. The cells were seeded into the collagen I-coated 96 well culture plate (Iwaki, Tokyo, Japan) at a density of 5 × 10^4^ cells/well with the seeding medium for 4 hours, then maintained in the culture medium of Power Primary HEP medium (Takara Bio, Shiga, Japan). The cells were cultured 5 - 7 days before treatment with test articles.

### In-cell ELISA

A previously described ELISA method [22] was applied to HepaSH cells with some modifications. Culture medium in 96 well plates was discarded, 100 μL of the test article dilution was added to each well and incubated at 37◦C under the 5% CO2 atmospheres for 0.5–48 hours. The test article solutions were discarded, washed with PBS, fixed with 4% paraformaldehyde phosphate buffer solution at room temperature for 10 minutes, washed with and phosphate buffer saline (PBS), permeabilized with 99.5% ethanol, tightly sealed and kept at − 20 ◦C until further use. Then, the ethanol was discarded and the plates were blocked with 1% bovine serum albumin (BSA, Nacalai Tesque, Kyoto, Japan) solution in PBS for 1 h at room temperature. After removal of the blocking solution, the plates were incubated for 2 h at room temperature with an Alexa 488 conjugated anti-γH2AX antibody (Anti-γH2AX phosphor S139, EP854 [2]Y ab195188, Abcam, Cambridge, UK) diluted 1/1000 in PBS with 1% BSA. The plates were subsequently washed three times with PBS and incubated for 15 minutes at room temperature with 50 μL/well 4’,6-diamidino-2-phenylindole (DAPI, Fujifilm) solution at a concentration of 1 μg/ml in PBS to measure DNA contents. Fluorescence for γH2AX (filter set, ex0.485 nm, em0.535 nm) and for DNA (ex0.360 nm, em0.465 nm) were measured with a microplate reader (TECAN Infinit 200 Pro, Tecan Japan, Kanagawa, Japan). Fluorescence intensity (FI) of each well was calculated by subtraction of mean measurement values of blank wells. FI for γH2AX was adjusted using FI of DAPI in the same well. γH2AX index was calculated,

$${\rm{\gamma H2AX}}\;{\rm{index}}\;{\rm{ = }}\;{{{\rm{FI}}\;{\rm{\gamma H2AX}}\;{\rm{in}}\;{\rm{treated}}\;{\rm{well}}{\rm{/}}{\rm{FI}}\;{\rm{DAPI}}\;{\rm{in}}\;{\rm{treated}}\;{\rm{well}}} \over {{\rm{FI}}\;{\rm{\gamma H2AX}}\;{\rm{in}}\;{\rm{control}}\;{\rm{well}}{\rm{/}}{\rm{FI}}\;{\rm{DAPI}}\;{\rm{in}}\;{\rm{control}}\;{\rm{well}}}}$$

Relative FI of DAPI in test wells to control wells were used for cell survival percentage and for normalization of FI γH2AX. Each experiment was carried out in duplicate and the mean FI for each treatment was calculated.

### Microscopy

Cells stained in the ELISA plates were microscopically observed with ZOE fluorescent cell imager (BioRad, Hercules, CA).

## Results

### γH2AX index of non-treated cells

**Fig. 1:**
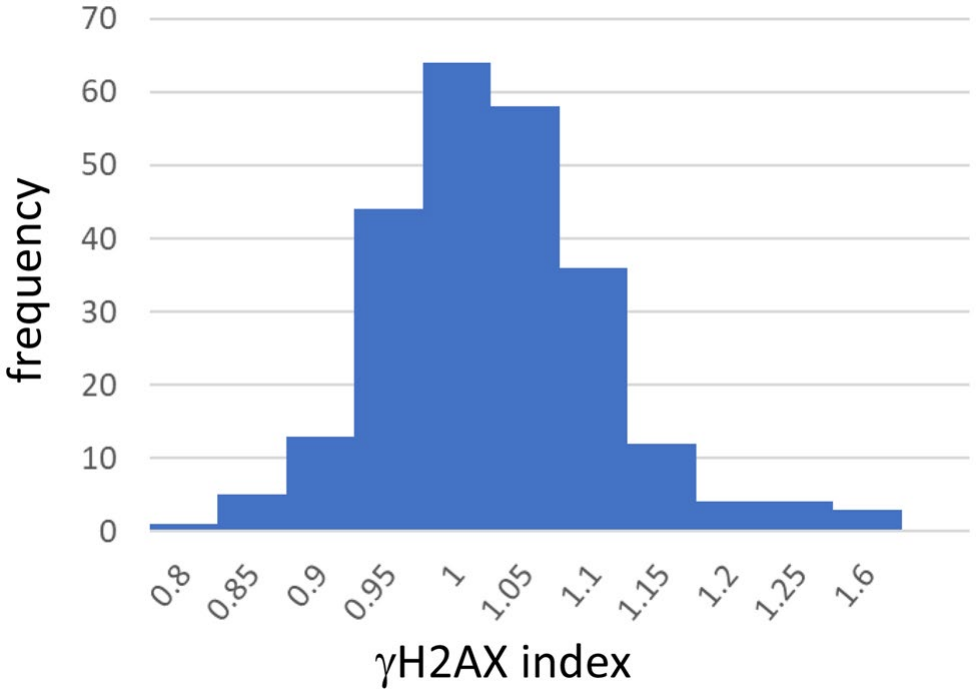
Histogram of γH2AX indices of 246 control wells. The data included indices of different production lots of three strains of HepaSH cells. The range of 95 percentile was from 0.85 to 1.22

The γH2AX indices of 246 untreated culture wells containing HepaSH cells were presented as a histogram (Fig. 1). Data were collected over a 9-month period, encompassing 11 lots of HepaSH cells of the three strains. The mean and standard deviation of the γH2AX index were 1.00 and 0.09, respectively. The observed γH2AX indices ranged from 0.78 to 1.57. After excluding the upper and lower 2.5% of the data – specifically, the cutoff points at 1.227 (the 6^th^ highest value, upper 2.4%) and 0.847 (the 6^th^ lowest value, lower 2.4%) out of the 246 data points - the 95th percentile range was determined to be between 0.85 and 1.22. Based on this distribution, we defined a γH2AX index greater than 1.2 as the threshold for a positive result in this study.

### Response to non-mutagenic compounds

**Fig. 2:**
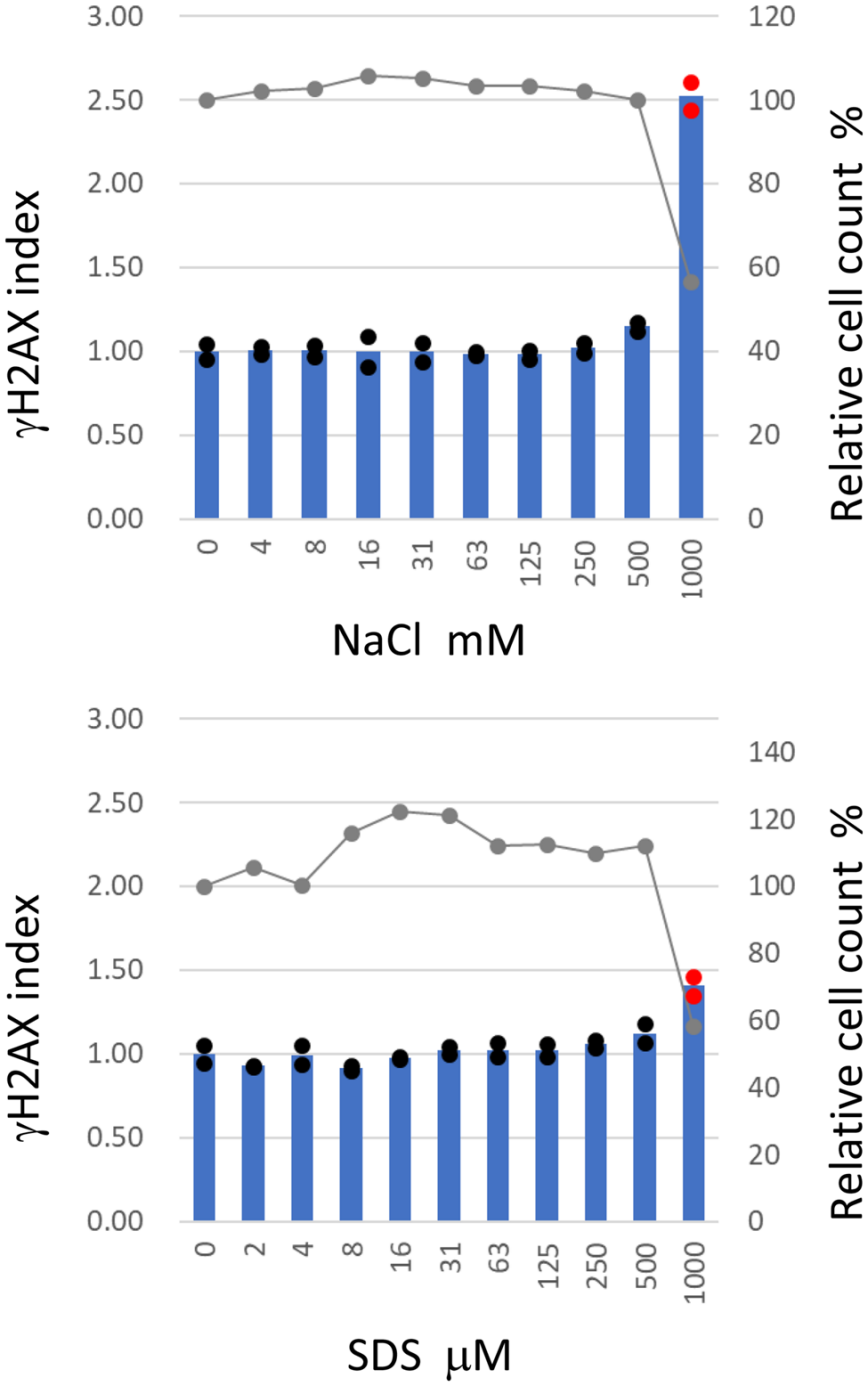
γH2AX index of HepaSH-E cells treated with non-genotoxic test compounds after 24 hours exposure. Blue bars (left vertical axis) indicate the mean gH2AX index from duplicate wells. Circles represent the index of each individual culture well: red indicates values exceeding the positive criteria, black indicates values within the background negative range. Gray lines (right vertical axis) represent the mean relative cell survival

Treatment with SDS and sodium chloride for 24 hours resulted in similar dose–response curves for γH2AX indices in HepaSH-E cells. The index increased only at the highest concentrations, where severe cytotoxicity was observed (Fig. 2). At doses where cell viability dropped below 60%, γH2AX indices exceeded the positive threshold of 1.2. At concentrations without severe cytotoxicity, γH2AX indices remained within the 95th percentile range of the background. Consistent γH2AX induction at highly cytotoxic doses of SDS or sodium chloride was also confirmed in reproducible experiments.

### Response to direct mutagens

**Fig. 3:**
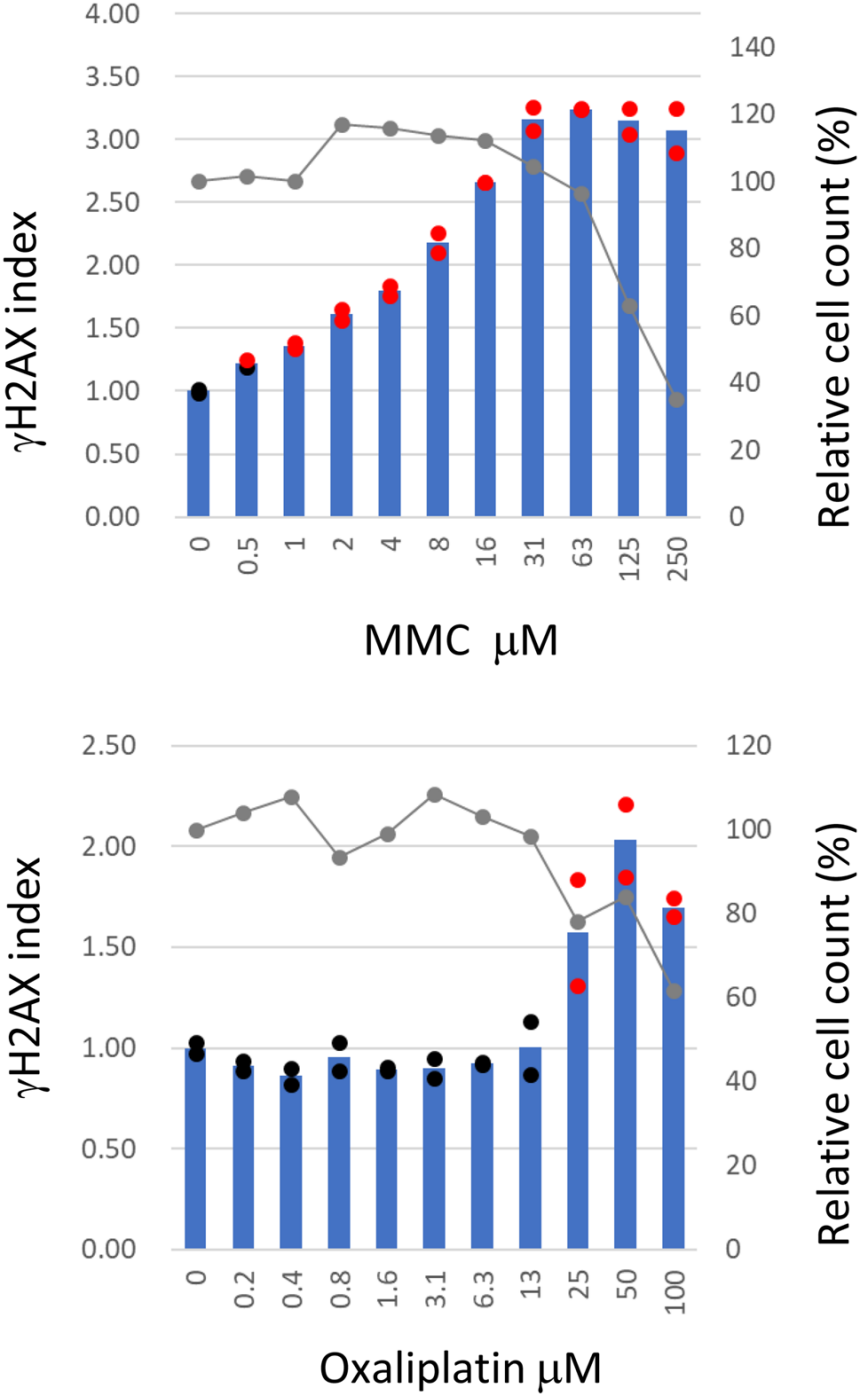
Dose dependent increase of γH2AX index of HepaSH-E cells treated with direct mutagens after 24 hours exposure. Blue bars (left vertical axis) indicate the mean gH2AX index from duplicate wells. Circles represent the index of each individual culture well: red indicates values exceeding the positive criteria, black indicates values within the background negative range. Gray lines (right vertical axis) represent the mean relative cell survival

Figure 3 illustrates the dose-dependent increase in γH2AX indices in HepaSH-E cells following 24-hour exposure to either MMC or oxaliplatin. MMC elevated the γH2AX index at concentrations of 1 μM and above in both duplicate wells, with the highest index observed at 63 μM, where minimal cytotoxicity was detected. Oxaliplatin treatment for 24 hours resulted in a γH2AX index exceeding the positive threshold at doses of 25 μM and higher in the duplicate wells. At 100 μM, cell viability following oxaliplatin exposure was 62%.

### Time course of induction of γH2AX with BaP

**Fig. 4:**
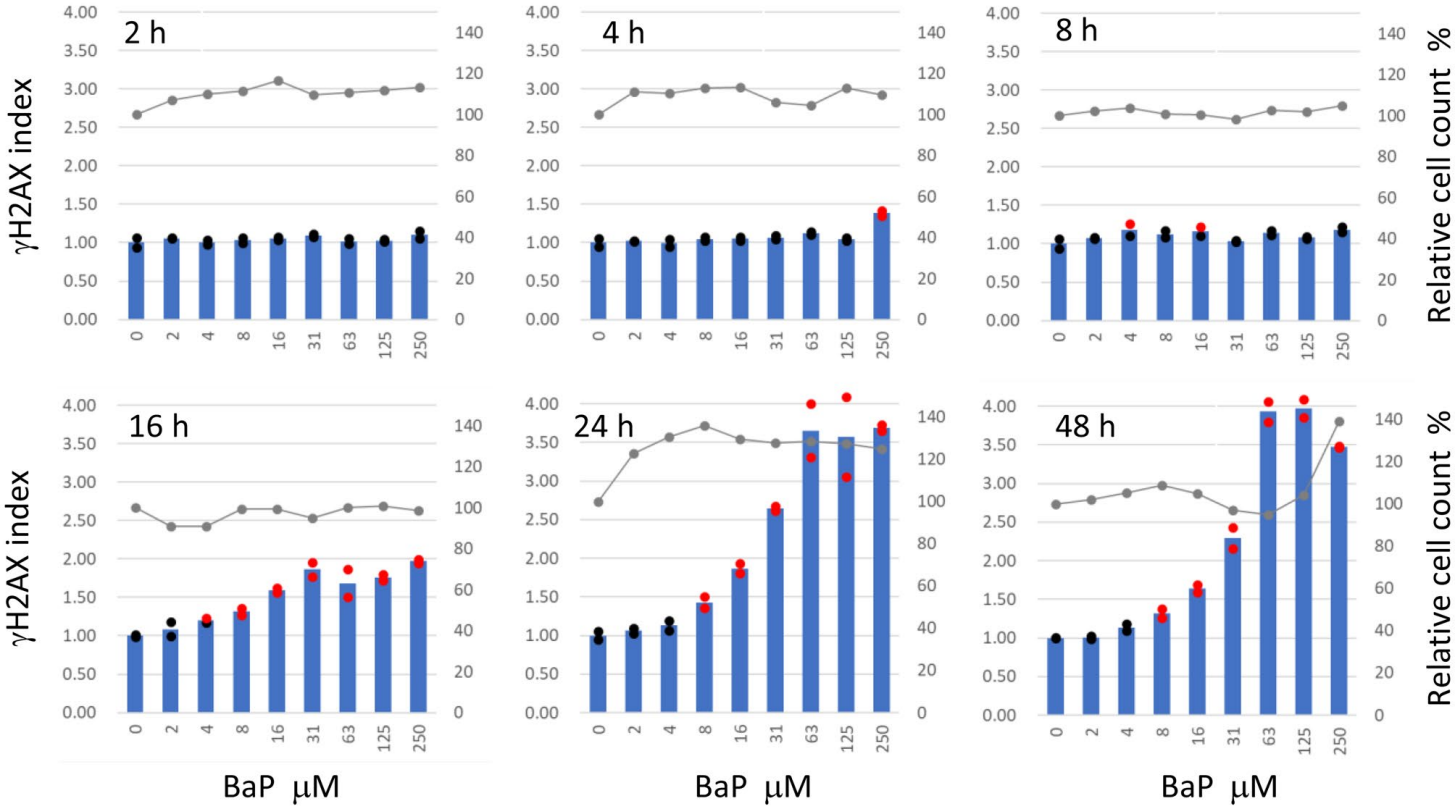
Time course of induction of γH2AX in HepaSH-E cells exposed to BaP. Blue bars (left vertical axis) indicate the mean gH2AX index from duplicate wells. Circles represent the index of each individual culture well: red indicates values exceeding the positive criteria, black indicates values within the background negative range. Gray lines (right vertical axis) represent the mean relative cell survival. Slight precipitation of the test compound was seen at 250 μM

**Fig. 5:**
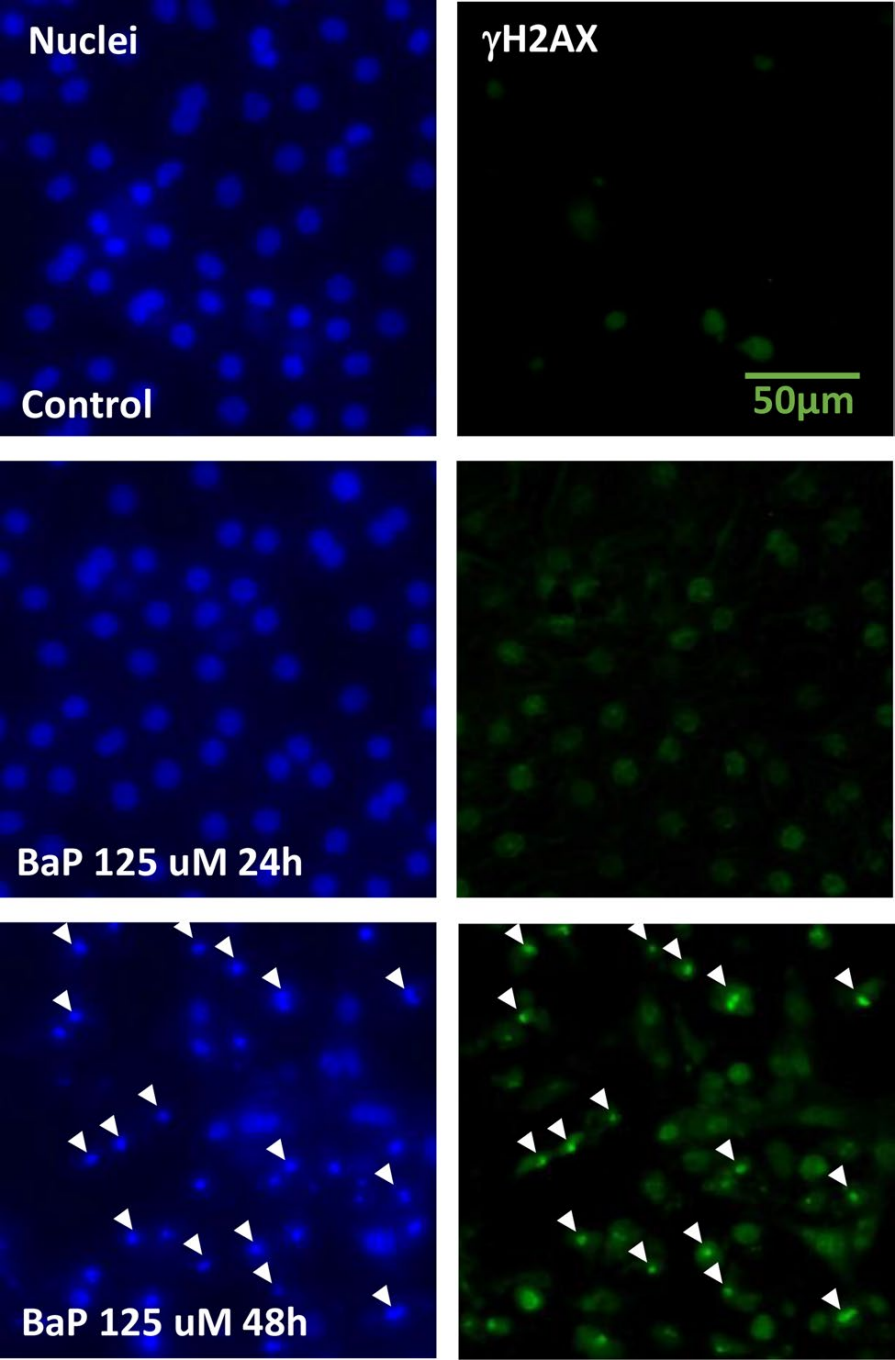
Microscopic observation of Hepa SH-E cells in in cell ELISA plates after treatment with Bap for 24 or 48 hours. HepaSH cells stained with DAPI on the left column and Alexa488 labeled anti-γH2AX antibody on the right. There were shrunk nuclei with condensed green fluorescent (arrows) after 48 hours treatment with 125 μM BaP

Induction of γH2AX in HepaSH-E cells was observed at 2, 4, 8, 16, 24, and 48 hours following treatment with BaP (Fig. 4). Slight precipitation of the test compound was noted at 250 μM. A dose-dependent increase in γH2AX indices was evident after 16 hours or longer exposure. The lowest concentrations that induced a positive γH2AX index in duplicate wells were 8 μM at 16 and 24 hours, and 4 μM at 48 hours. Peak index values increased in a time-dependent manner from 16 to 48 hours. Cells in the ELISA assay culture plates were also examined microscopically (Fig. 5). Several γH2AX-positive cells with green fluorescence were observed in control wells, while numerous positive cells were seen in wells treated with 125 μM BaP for 24 and 48 hours. In the 48-hour treated wells, cells with shrunken nuclei and condensed green fluorescence were observed, which were not present in the 24-hour treated wells.

### Influence of DMSO concentration on induction of γH2AX after treatment with BaP

**Fig. 6:**
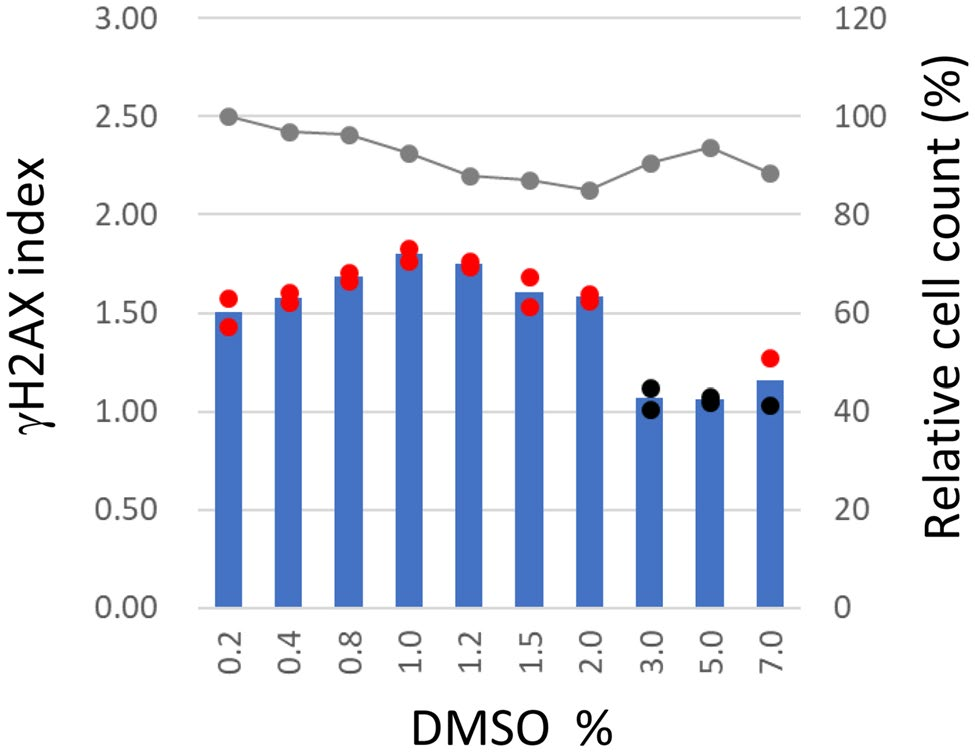
Reduction of γH2AX index with 3% or higher DMSO concentrations in culture wells. HepaSH-E cells were exposed to 200 μM BaP together with DMSO. Blue bars (left vertical axis) indicate the mean gH2AX index from duplicate wells. Circles represent the index of each individual culture well: red indicates values exceeding the positive criteria, black indicates values within the background negative range. Gray lines (right vertical axis) represent the mean relative cell survival

DMSO reduced γH2AX responses following BaP treatment in HepaSH-E cells (Fig. 6). Cells were exposed to 200 μM BaP in the presence of 0.2–7.0% DMSO in the culture medium for 24 hours. No significant cytotoxicity was observed at any DMSO concentration, with cell viability remaining at 85% or higher. Mean γH2AX indices ranged from 1.50 to 1.80 at DMSO concentrations between 0.2% and 2.0%. When the DMSO concentration reached 3.0% or higher, mean γH2AX indices fell within the negative range, below 1.20, though a positive value was seen in 1/2 well at 7.0% DMSO.

### Reproducibility of induction of γH2AX with BaP

**Fig. 7:**
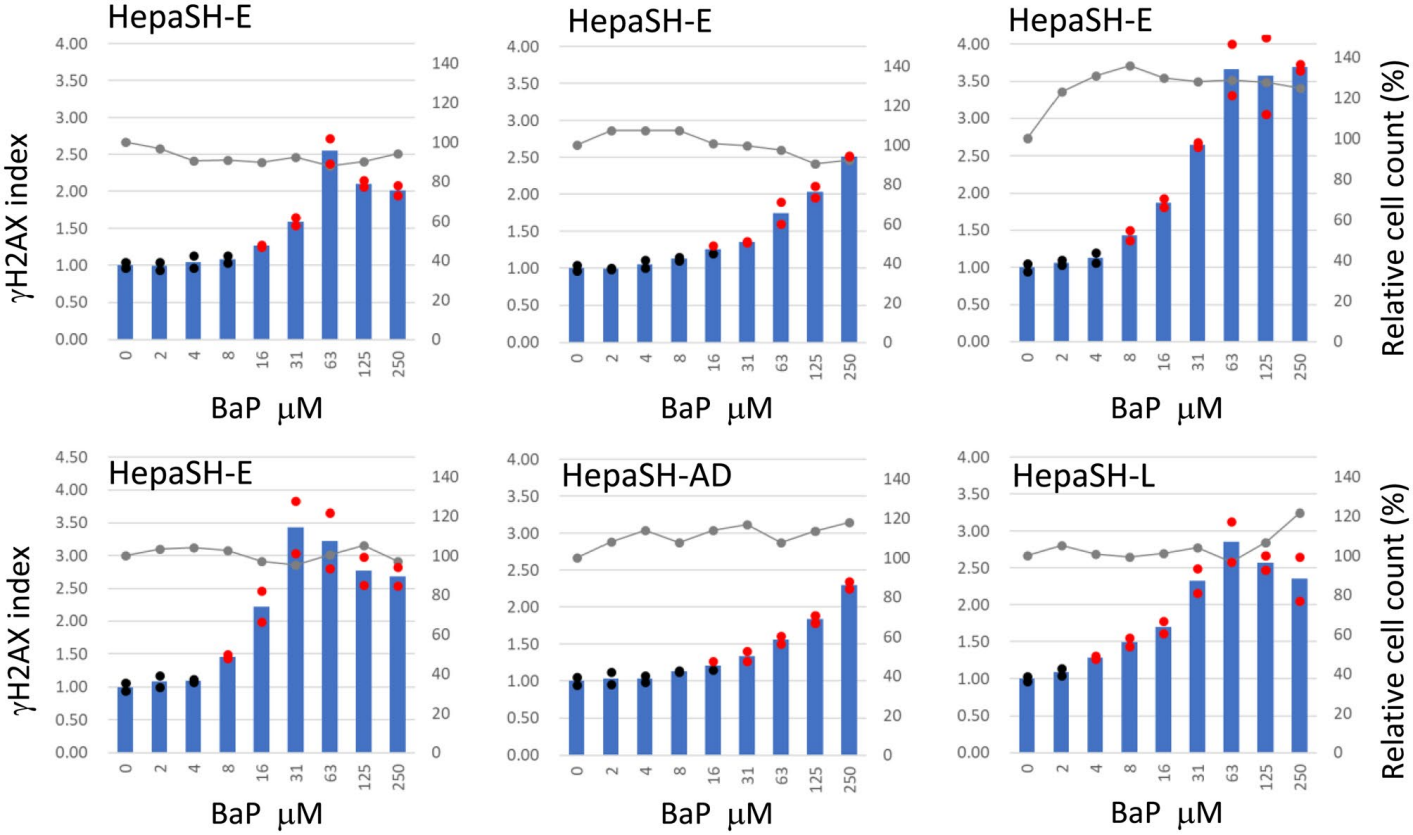
Reproducible dose related increase of γH2AX index of HepaSH cells from three donors after treatment with Bap for 24 hours. Tests with HepaSH-E were repeated on different days. Blue bars (left vertical axis) indicate the mean gH2AX index from duplicate wells. Circles represent the index of each individual culture well: red indicates values exceeding the positive criteria, black indicates values within the background negative range. Gray lines (right vertical axis) represent the mean relative cell survival. Slight precipitation of test compound was seen at 250 μM

Figure 7 shows the induction of γH2AX following 24-hour BaP treatment in three different HepaSH cell strains across multiple test days. BaP induced a dose-dependent increase in γH2AX levels in all the three strains. The minimum concentrations required to generate a positive γH2AX index were 8 or 16 μM for HepaSH-E, 16 μM for HepaSH-AD, and 4 μM for HepaSH-L. The peak mean γH2AX index values were 2.52–3.69 in HepaSH-LE, 2.30 in HepaSH-AD, and 2.84 in HepaSH-L.

### Induction of γH2AX with AA or PhIP.

**Fig. 8:**
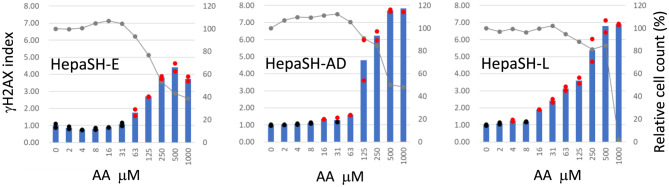
Dose dependent increase of γH2AX index of HepaSH cells from three donors after treatment with aristolochic acid for 24 hours. Blue bars (left vertical axis) indicate the mean gH2AX index from duplicate wells. Circles represent the index of each individual culture well: red indicates values exceeding the positive criteria, black indicates values within the background negative range. Gray lines (right vertical axis) represent the mean relative cell survival

**Fig. 9:**
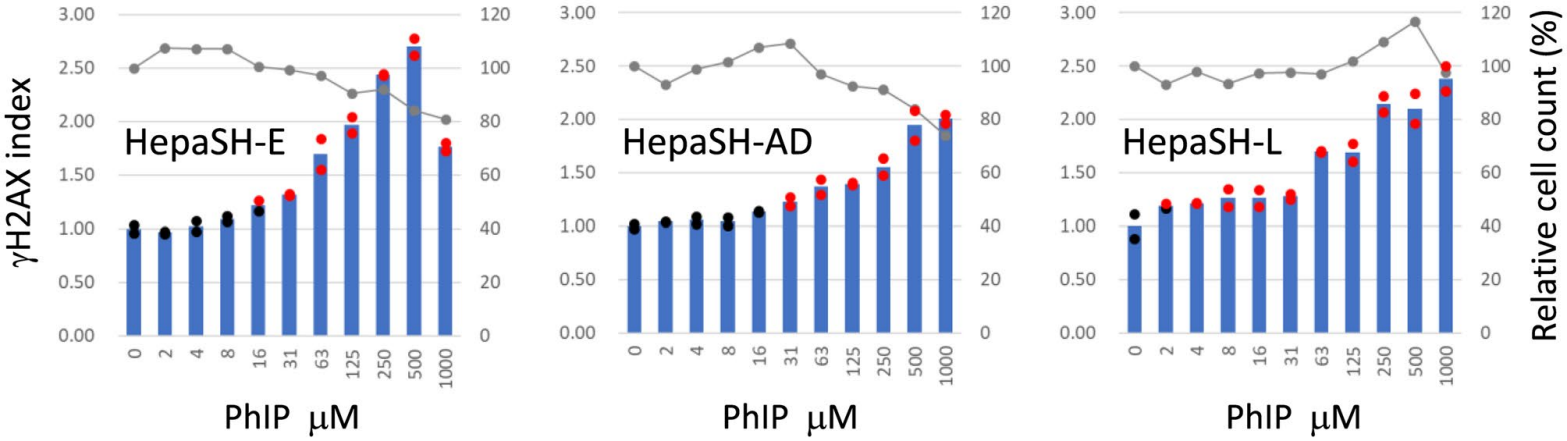
Dose dependent increase of γH2AX index of HepaSH cells from three donors treated with PhIP after 24 hours exposure. Blue bars (left vertical axis) indicate the mean gH2AX index from duplicate wells. Circles represent the index of each individual culture well: red indicates values exceeding the positive criteria, black indicates values within the background negative range. Gray lines (right vertical axis) represent the mean relative cell survival

Dose-dependent induction of γH2AX was observed in cells of all the three strains following 24-hour treatment with AA (Fig. 8) or PhIP (Fig. 9). After AA treatment (Fig. 8), stronger γH2AX induction was seen in HepaSH-AD and HepaSH-L cells compared to HepaSH-E. In contrast, following PhIP treatment (Fig. 9), γH2AX induction appeared weaker in HepaSH-AD cells than in HepaSH-E and HepaSH-L.

## Discussion

In this study, we developed an assay system to measure γH2AX levels in HepaSH cells -stable human hepatocytes- using in-cell ELISA (γH2AX-SHE). A dose dependent increase in γH2AX levels following treatment with MMC and oxaliplatin (Fig. 3) demonstrated that γH2AX is a useful DNA damage marker in non-proliferating HepaSH cells. γH2AX was initially identified as a sensitive marker of DNA double strand breaks (DSB) following exposure to ionizing radiation and was later found to be induced by single strand breaks or base and backbone modifications, primarily through replication fork collapse or DNA repair processes resulting in DSBs [23]. Subsequent studies have supported that γH2AX is induced by variety of DNA-damaging agents [24, 25]. Since HepaSH cells were non-proliferative, γH2AX induction seen in this study is likely attributed to transcription coupled repair mechanisms.

Accumulated data from 246 negative control wells of HepaSH cells derived from three donors showed γH2AX index ranging from 0.78 to 1.57 (Fig. 1). By excluding the upper and lower 2.5% of the distribution, the 95-percentile range was determined to be 0.85 to 1.22. We consequently established a positive threshold for the γH2AX index at 1.20, categorizing values exceeding this threshold as indicative of a DNA damage response.

The assay elicited the dose dependent increase of γH2AX levels following treatment with BaP, AA or PhIP (Figs. 7, 8, 9). Key human metabolic enzymes responsible for generating genotoxic metabolites of BaP, AA and PhIP are cytochrome P450 (CYP) 1A1 [26, 27], NAD(P)H:quinone oxidoreductase (NQO1) [28, 29] and CYP1A2 [30, 31], respectively. The positive genotoxic responses observed with BaP, AA and PhIP in this study indicate that these human metabolic enzymes effectively activated the test articles to cause DNA damages in HepaSH cells, which was successfully using the γH2AX-SHE assay. These findings suggest that γH2AX-SHE is a helpful tool for a genotoxicity assessment in systems that mimic human metabolic activation.

Non-genotoxic agents exhibited “hockey stick” dose-response curves of γH2AX indices in this study (Fig. 2). Although the cause for the excessive elevation of γH2AX index at the highly cytotoxic concentrations remains unclear, previous reports described that DNA doble strand breaks were formed by non-mutagenic, non-carcinogenic compounds under highly cytotoxic conditions [32], which might contribute to γH2AX induction. Positive responses only at highly cytotoxic concentration ranges are considered biologically irrelevant in current standard in vitro cytogenetic assays using mammalian cells [33–35]. Based on the results of this study, positive responses observed in γH2AX-SHE at cell survival rates below 60% are deemed biologically irrelevant. The cutoff aligns with criteria used in the in vitro micronucleus test and chromosomal aberration test where 50% reduction in population doubling is target as the cytotoxicity threshold at the highest treatment concentration [1, 4]. Because the cells are damaged before dis-attachment from the surface of culture plate, DAPI-based measurements of cell numbers may lead underestimation of cytotoxicity. Additional cytotoxicity parameters of an intracellular or released enzymatic activity assay can provide more useful information for biological relevance.

BaP did not elicit a positive response after 8 hours or shorter exposure, but became positive after 16 hours (Fig. 4). In standard in vitro genotoxicity tests using mammalian cells with rat liver S9, the recommended treatment duration is typically 3 to 6 hours. In these assays, test compounds can directly interact with metabolic enzymes when added to culture wells containing liver S9. In contrast, the γH2AX-SHE system requires test compounds to pass through the cell membrane before encountering intracellular metabolic enzymes. This difference partly explain why γH2AX-SHE requires longer exposure periods to detect genotoxic responses induced by BaP.

We consider 24-hour treatment to be an appropriate experimental condition for the γH2AX-SHE. Dose dependent increase in γH2AX levels were consistently observed after 24-hour exposure to both direct and indirect genotoxic compounds in this study (Figs. 3, 4, 7, 8 and 9). Although BaP treatment resulted in slightly higher γH2AX measurements after 48 hours compared to 24 hours (Fig. 4), we do not recommend the 48-hour treatment. Shrunken nuclei exhibiting condensed green fluorescence for γH2AX were frequently observed after 48-hour treatment but not at 24 hours (Fig. 5). Nuclear pyknosis is a morphological hallmark of apoptosis triggered by genotoxic stress [36–39]. Since dying cells are not considered a direct cause of carcinogenesis, we propose that excluding apoptotic cells is preferable when applying γH2AX as a biomarker for genotoxic hazard assessment.

Regarding preincubation and statistic evaluation, we selected a 5- to 7-day preculture period, which consistently yielded positive responses following treatment with BaP, AA or PhIP. Under the in vitro conditions, HepaSH cells maintained cell viability, albumin secretion and experimentally relevant CYP-dependent drug-metabolizing activity for up to 21 days [20]. To define the positive criterion – exceeding a γH2AX index of 1.2 - we did not account for individual strain differences, as the control data from HepaSH-AD and -E were insufficient to establish strain-specific thresholds. Further investigation is needed to establish a more appropriate positive threshold and statistical approach for decision making.

Induction of γH2AX by BaP was inhibited at DMSO concentrations of 3% or higher in culture wells (Fig. 6). In contrast, DMSO at concentrations of 2% or lower did not suppress γH2AX induction by BaP. We propose that DMSO concentrations should not exceed 1% because influence of DMSO on various enzymatic activities remain unclear in HepaSH cells. The use of 1% DMSO is well established in standard genotoxicity tests including the in vitro mammalian cell micronucleus test [4], chromosomal aberration test [1] and thymidine kinase gene mutation test [2].

A dose-dependent increase in γH2AX induced by BaP was consistently reproduced in HepaSH cell strains derived from three donors (Fig. 7). Although the lowest positive doses and peak γH2AX levels varied across experiments, the overall dose response curve shapes of HepaSH-AD and HepaSH-L appeared to fall within the range of variation observed in four repeated experiments using HepaSH-E. Strain-specific differences were also observed following treatment with AA or PhIP (Figs. 8, 9). These variations may reflect individual donor responses. Uehara et al. [20] observed similar expression levels of nine CYP genes among five HepaSH strains. However, strains derived from donors with poor metabolizer genotype of CYP2C19 or CYP3A4 exhibited markedly lower metabolic activities of those enzymes. A later study [40] reported higher CYP1A2 activity in HepaSH-L compared to HepaSH-E and -AD, which appeared consistent with the γH2AX induction observed following PhIP treatment. Notably, PhIP—activated by CYP1A2—induced γH2AX at lower doses in HepaSH-L than in HepaSH-E or -AD (Fig. 9). Zerdoug et al. [21] demonstrated CYP induction in HepaSH cells after exposure to environmental chemicals. The observed variation among HepaSH strains may reflect differences in genetic polymorphisms or enzyme inducibility. However, a slightly larger difference was noted in the duplicate control wells of HepaSH-L than HepaSH-E or -AD following PhIP treatment (Fig. 9), and further confirmation is needed to clarify the extent of strain variability.

HepG2 and HepaRG cells have been widely used in in vitro experiments designed to mimic human liver metabolism. HepaRG cells, derived from a liver tumor, have been proposed as a human-relevant in vitro genotoxicity test system due to their expression of various Phase I and Phase II metabolic enzymes [41]. Compared to HepG2 cells, HepaRG cells have shown greater sensitivity in detecting the genotoxic potential of indirect mutagens [42]. Comet, micronucleus and γH2AX assays using HepaRG cells successfully indicated positive responses to indirect genotoxic chemicals [43–47]. In contrast, HepaSH cells are non-proliferative, making comet and micronucleus assays inapplicable. However, γH2AX assays remain a sensitive method for detecting DNA damage. HepaSH strains are derived from different donors, and new strains can be readily added. This flexibility offers advantages for research aimed at elucidating inter-individual variability and for safety assessments that require coverage of a diverse human population.

## Conclusions

The γH2AX-SHE assay can serve as a useful tool for detecting DNA damage under conditions that mimic human metabolic activity. Based on the study results, the following recommendations were made: a 24-hour treatment period, a DMSO concentration not exceeding 1%, and recognition that positive responses observed at highly cytotoxic doses - defined as roughly less than 60% cell survival - may be biologically irrelevant.

## Data Availability

No datasets were generated or analysed during the current study.

## Abbreviations

AA: Aristolochic acid
BaP: Benzo(a)pyrene
CYP: Cytochrome P450
DAPI: 4’,6-diamidino-2-phenylindole
DMSO: Dimethylsulfoxide
FI: Fluorescence intensity
γH2AX-SHE: γH2AX in stable human hepatocytes ELISA
MMC: Mitomycin C
PhIP: 2-amino-1-methyl-6-phenylimidazo(4,5-b)pyridine
SDS: Sodium dodecyl sulfate
