# Correction: Development of a DNA damage assay system using stable human hepatocytes

**DOI:** 10.1186/s41021-026-00355-1

**Published:** 2026-03-02

**Authors:** Masayuki Mishima, Kazuki Izawa, Masataka Tsuda, Yuichiro Higuchi, Shotaro Uehara, Hiroshi Suemizu, Kei-Ichi Sugiyama

**Affiliations:** 1 Division of Genome Safety Science, National Institute of Health Sciences (NIHS), 3-25-26 Tonomachi, Kawasaki-ku, Kawasaki, Kanagawa 210-9501 Japan; 2 Liver Engineering Laboratory, Department of Research for Humanized Model, Central Institute for Experimental Medicine and Life Science (CIEM), 3-25-12 Tonomachi, Kawasaki-ku, Kawasaki, Kanagawa 210-0821 Japan

**Correction: Genes and Environment (2026) 48:3**

10.1186/s41021-025-00347-7

Following publication of the original article [1], we were notified that of an error in the unit for the concentration of MMC in Fig. 3, which was incorrectly stated as nM instead of the correct unit µM.

The original article has been corrected.
